# NDM-Producing *Escherichia coli* subdural empyema mimicking chronic subdural hematoma: a case report

**DOI:** 10.3389/fmed.2026.1930920

**Published:** 2026-09-23

**Authors:** Xinlei Wang, Jiayue Li, Yantong Zhuge, Ran Chen, Qi Gao, Yilin Zhao, Qipeng Jin, Yiyi Zhang

**Affiliations:** Department of Critical Care Medicine, Longhua Hospital, Shanghai University of Traditional Chinese Medicine, Shanghai, China

**Keywords:** chronic subdural hematoma, escherichia coli, metagenomic next-generation sequencing, New Delhi metallo-*β*-lactamase, source control, subdural empyema

## Abstract

**Background:**

Subdural empyema is a life-threatening intracranial infection that may mimic chronic subdural hematoma on non-contrast computed tomography (CT), delaying diagnosis and source control. Intracranial infection caused by New Delhi metallo-*β*-lactamase (NDM)-producing *Escherichia coli* is rare and may be overlooked when bloodstream infection appears microbiologically controlled.

**Case presentation:**

An 82-year-old man presented with fever and progressive neurological deterioration. Chest computed tomography (CT) suggested right lower lobe pneumonia, whereas serial head CT scans showed a left frontotemporoparietal subdural collection that was repeatedly interpreted as chronic subdural hematoma. Blood culture yielded multidrug-resistant *Escherichia coli*, and blood metagenomic next-generation sequencing (mNGS) detected *E. coli* together with *bla*NDM and other resistance determinants. Despite microbiologically guided antimicrobial therapy and subsequent clearance of blood cultures, fever, impaired consciousness, and enlargement of the subdural lesion persisted. Burr-hole exploration on March 22 revealed yellow purulent material rather than hematoma, establishing the anatomical diagnosis of subdural empyema and providing source control. Conventional culture of the surgically obtained subdural pus also yielded multidrug-resistant E. coli, while pus mNGS detected *E. coli*, *bla*NDM, *bla*CTX-M, and *mcr-1*. At the latest follow-up, the patient was undergoing further rehabilitation at a rehabilitation hospital. His vital signs were stable, and his Glasgow Coma Scale score was 15.

**Conclusion:**

Negative follow-up blood cultures do not exclude persistent compartmentalized intracranial infection. In bacteremic patients with neurological deterioration and an enlarging subdural collection, subdural empyema should be reconsidered and timely source control should not be delayed.

## Introduction

Subdural empyema is a life-threatening intracranial infection requiring prompt diagnosis and source control. It usually arises from contiguous sinus, otogenic, or postoperative infection, but hematogenous spread can also occur, particularly when a pre-existing subdural collection or hematoma cavity is present (1, 2). Diagnosis may be difficult when non-contrast computed tomography (CT) resembles chronic subdural hematoma. Contrast-enhanced magnetic resonance imaging (MRI) and diffusion-weighted imaging (DWI) can provide additional information by revealing inflammatory meningeal enhancement and diffusion abnormalities within purulent collections, features that are usually not well characterized on non-contrast CT (3–5).

*Escherichia coli* is an uncommon cause of adult subdural empyema, and intracranial infection caused by New Delhi metallo-*β*-lactamase (NDM)-producing strains is rare (6, 7). Identification of NDM has immediate therapeutic implications because infections caused by metallo-*β*-lactamase-producing Enterobacterales often require specialized antimicrobial strategies, including aztreonam with avibactam protection or cefiderocol when appropriate (8, 9). Metagenomic next-generation sequencing (mNGS) has also emerged as a useful adjunctive diagnostic tool for central nervous system (CNS) infections, particularly when conventional microbiological evidence is incomplete or delayed (10, 11). We report an elderly patient with multidrug-resistant *E. coli* bloodstream infection and detection of blaNDM by blood mNGS, in whom a progressive subdural lesion repeatedly mimicked chronic subdural hematoma on CT but was ultimately confirmed as subdural empyema. This case highlights the clinical and public-health importance of antimicrobial resistance, mNGS-supported diagnosis, and timely source control. An overview of the clinical course is provided in Figure 1.

**Figure 1:**
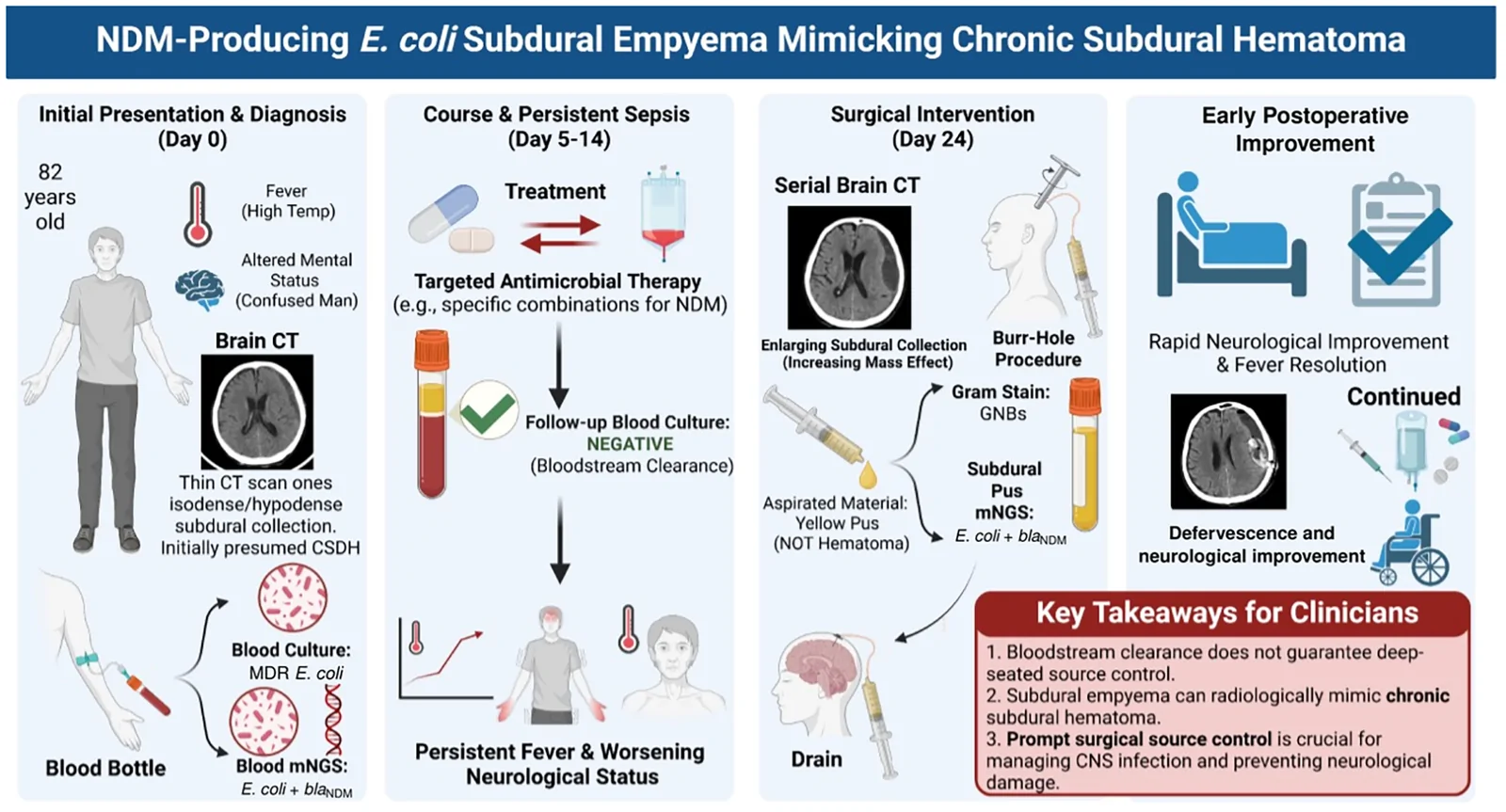
Graphical abstract of the clinical course. Blood culture yielded multidrug-resistant *Escherichia coli*, whereas blood mNGS detected *E. coli* and blaNDM. Despite antimicrobial therapy, fever, neurological deterioration, and enlargement of the subdural collection persisted. Surgical drainage confirmed subdural empyema and was followed by clinical improvement. Created in BioRender. Li, J. (2026) https://BioRender.com/zmpsizs.

## Case presentation

An 82-year-old man was admitted on February 26, 2026, with a 3-day history of fever peaking at 38.7 °C. He had undergone radical subtotal gastrectomy with gastroenteric anastomosis for gastric cancer in 2024, but adjuvant chemotherapy had been discontinued because of intolerance. Laboratory tests showed a C-reactive protein level of 270.80 mg/L, a procalcitonin level of 2.629 ng/mL, and a neutrophil count of 7.82 × 10⁹/L. Chest computed tomography (CT) suggested right lower lobe pneumonia. Non-contrast head CT showed a 13-mm heterogeneous left frontotemporoparietal subdural collection, initially interpreted as chronic subdural hematoma (Figure 2A).

**Figure 2:**
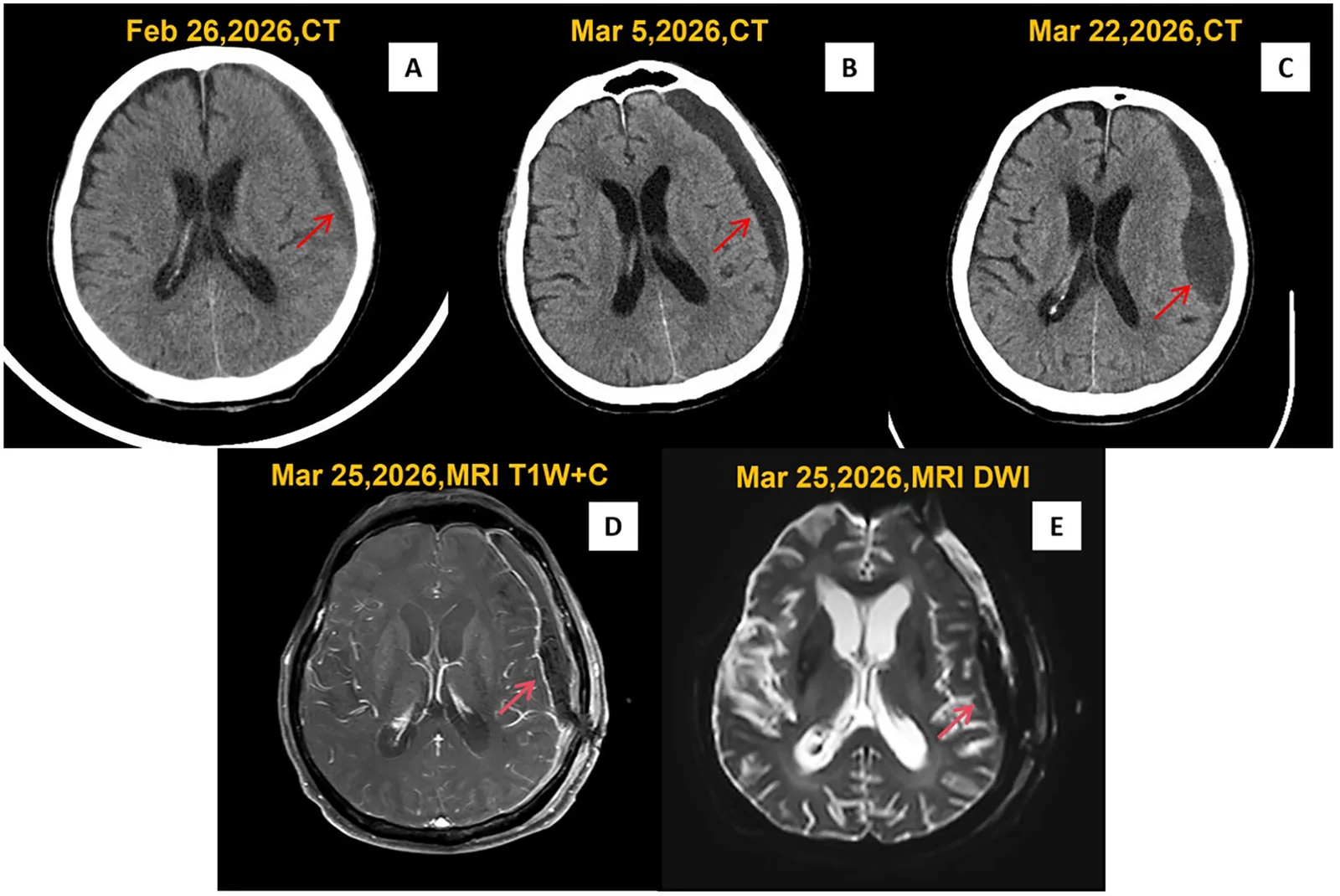
Serial neuroimaging evolution from presumed chronic subdural hematoma to surgically confirmed subdural empyema. **(A)** Non-contrast head CT on February 26, 2026, showed a 13-mm heterogeneous left frontotemporoparietal subdural collection, initially interpreted as possible chronic subdural hematoma. **(B)** Follow-up non-contrast CT on March 5 showed slight enlargement of the collection with decreased density. **(C)** Non-contrast CT on March 22, immediately before burr-hole exploration, showed enlargement of the lesion to 22 mm, with adjacent cerebral edema and sulcal effacement. **(D)** Postoperative contrast-enhanced T1-weighted MRI on March 25 showed subdural blood products, residual subdural fluid, and marked dural and arachnoid enhancement. **(E)** Diffusion-weighted imaging obtained during the same examination showed hyperintense signal within the residual collection, compatible with residual infectious or inflammatory material but not independent evidence of preoperative restricted diffusion.

Empirical ceftazidime–avibactam was initiated on admission. Because fever and neurological deterioration persisted, the patient was transferred to the intensive care unit on March 2 with a Glasgow Coma Scale score of 11, and antimicrobial therapy was escalated to meropenem after preliminary blood culture revealed gram-negative bacilli. Blood culture on March 4 identified multidrug-resistant *Escherichia coli*. Tigecycline was briefly added because a possible gastrointestinal source was considered in view of the patient's previous subtotal gastrectomy with gastroenteric anastomosis. On March 6, blood metagenomic next-generation sequencing detected *E. coli* and *bla*NDM, prompting a switch to aztreonam–avibactam combined with amikacin; amikacin was selected on the basis of the reported *in vitro* susceptibility. However, despite subsequent clearance of the bloodstream infection, fever and neurological impairment persisted. On March 13, amikacin was discontinued because of the inadequate clinical response, and eravacycline was added as combination therapy after PET-CT showed inflammatory changes around the gastroenteric anastomosis, further supporting a possible gastrointestinal source. At that time, the subdural collection was still interpreted as a chronic subdural hematoma rather than an intracranial infection. Impaired consciousness and radiological progression of the lesion nevertheless continued, indicating that microbiological clearance of the bloodstream infection had not achieved control of the intracranial focus.

Serial neuroimaging showed continued enlargement of the presumed chronic subdural hematoma. CT on March 5 demonstrated slight interval enlargement with decreased density (Figure 2B), while positron emission tomography–CT on March 13 did not clarify the nature of the lesion. Preoperative contrast-enhanced MRI and diffusion-weighted imaging were not obtained because the patient's critical condition made transport to the MRI suite unsafe. On March 22, repeat head CT showed enlargement of the subdural lesion to 22 mm, with adjacent cerebral edema and sulcal effacement (Figure 2C). Progressive lesion enlargement, persistent fever, and worsening consciousness prompted burr-hole exploration.

Intraoperatively, the dura was thickened, and approximately 30 mL of yellow purulent material rather than hematoma was encountered. The pus was evacuated, the cavity was irrigated, and a subdural drainage catheter was placed. Conventional pus culture yielded multidrug-resistant *E. coli*. The isolate was reported as resistant to colistin, with an MIC of ≥16, and susceptible to amikacin, minocycline, and tigecycline; however, the MIC unit, testing platform, and interpretive standard were not provided. Pus mNGS detected *E. coli*, *bla*NDM, *bla*CTX-M, and *mcr-1*. Because isolate-level molecular testing was not performed, these resistance genes could not be definitively assigned to the cultured isolate.

After surgical drainage, intravenous fosfomycin was added on March 23 as part of postoperative combination antimicrobial therapy because cerebrospinal fluid penetration has been documented. The patient became afebrile within 3 days, and his consciousness improved markedly by postoperative day 2. Postoperative MRI findings are shown in Figures 2D,E. Aztreonam–avibactam was continued until May 22, and at the latest follow-up, the patient was undergoing further rehabilitation at a rehabilitation hospital. His vital signs were stable, and his Glasgow Coma Scale (GCS) score was 15. Microbiological findings, the clinical timeline, and the detailed antimicrobial treatment course are summarized in Supplementary Tables S1–S3, respectively.

Surgical exploration revealed yellow purulent material and established the diagnosis of subdural empyema. The serial imaging findings illustrate how subdural empyema may mimic chronic subdural hematoma on non-contrast CT, with the anatomical diagnosis established only during surgical exploration.

## Discussion

### Possible hematogenous dissemination

This case supports hematogenous dissemination as the most plausible route of intracranial infection. Multidrug-resistant *Escherichia coli* was recovered from both blood and subdural pus, while mNGS detected *E. coli* and concordant resistance determinants in both specimens. Although the primary source of bacteremia could not be definitively established, there was no clear history of cranial trauma, neurosurgery, sinusitis, or otogenic infection. The temporal sequence was therefore consistent with secondary seeding of the subdural space after bloodstream invasion, as described in previous reports of *E. coli* infection involving pre-existing subdural collections (1, 6, 7). However, isolate-level PCR, whole-genome sequencing, or another strain-level comparison was not performed; therefore, clonal identity and the precise route of dissemination could not be definitively established.

### Diagnostic challenge and imaging limitations

The case also illustrates how subdural empyema may mimic chronic subdural hematoma on serial non-contrast CT. In an elderly patient, a hypodense or heterogeneous extra-axial collection can reasonably suggest chronic subdural hematoma at presentation. Nevertheless, persistent fever, neurological decline, multidrug-resistant *E. coli* bacteremia, and progressive lesion enlargement were inconsistent with an uncomplicated chronic subdural process. Earlier contrast-enhanced MRI and diffusion-weighted imaging were not obtained because the patient's critical condition made transport to the MRI suite unsafe. Repeated CT interpretations favored chronic subdural hematoma, while the fever was initially attributed to pneumonia and bloodstream infection, contributing to diagnostic anchoring. In retrospect, chronic subdural hematoma, subdural hygroma, and subdural empyema should all have been considered. Postoperative MRI findings were compatible with residual infectious or inflammatory material; however, because the examination was performed after surgery, they could not establish whether preoperative restricted diffusion had been present. Earlier preoperative MRI with diffusion-weighted imaging might have helped distinguish empyema from chronic hematoma or hygroma before surgical exploration (3–5).

### Persistent intracranial focus despite bloodstream clearance

A further management lesson is that negative follow-up blood cultures do not prove control of a deep-seated intracranial focus. In this patient, bloodstream infection appeared microbiologically improved after antimicrobial adjustment, but fever, inflammatory activity, neurological deterioration, and radiological progression persisted. The subdural space likely functioned as a compartmentalized infectious reservoir. This distinction is clinically important because negative follow-up blood cultures may create false reassurance when anatomical source control remains inadequate, particularly in infections caused by resistant Enterobacterales (8, 9, 12). In patients with persistent systemic inflammation, neurological decline, or radiological progression, clinicians should actively reconsider an unresolved deep-seated focus rather than assuming that bloodstream clearance reflects definitive infection control. The corresponding laboratory trends are shown in Supplementary Figure S1.

### Antimicrobial strategy

Aztreonam–avibactam combined with amikacin was selected after blood mNGS detected *bla*NDM. Aztreonam is stable to hydrolysis by metallo-*β*-lactamases, while avibactam protects it against co-produced serine *β*-lactamases; amikacin was added on the basis of the reported *in vitro* susceptibility (8, 9). Tigecycline and eravacycline were administered as combination therapy for the multidrug-resistant systemic infection, with a possible gastrointestinal source considered because of the patient's previous gastrectomy and PET-CT findings around the gastroenteric anastomosis. During both treatment periods, the subdural lesion was still interpreted as chronic subdural hematoma. Standard-dose tigecycline has shown low and variable cerebrospinal fluid exposure, whereas evidence regarding eravacycline exposure in the central nervous system remains largely preclinical; therefore, neither agent should be interpreted as definitive treatment for established subdural empyema (13, 14). Intravenous fosfomycin was added after drainage because cerebrospinal fluid penetration has been documented (15). However, cerebrospinal fluid penetration does not establish adequate exposure within a subdural empyema, and the postoperative improvement should be attributed to surgical source control combined with antimicrobial therapy rather than to fosfomycin alone (12).

### Role of surgical source control

Surgical drainage was the decisive turning point. Blood and pus mNGS provided etiological information and identified clinically relevant resistance determinants, but did not establish that the radiologically identified subdural collection was an empyema. The anatomical diagnosis was established when burr-hole exploration revealed purulent material, while evacuation, irrigation, and drainage provided the necessary source control. The rapid postoperative response, including defervescence and improved consciousness, supports the central role of source control in intracranial infection (12). The case therefore links antimicrobial-resistance management with a broader public-health message: microbiological clearance in blood should not create false reassurance when an uncontrolled, localized infectious focus remains clinically active.

### Limitations

This report has several limitations. First, it describes a single patient, and the primary source of bacteremia could not be definitively established. Second, although multidrug-resistant *Escherichia coli* was recovered from both blood and subdural pus and mNGS detected concordant resistance determinants in both specimens, isolate-level PCR, whole-genome sequencing, or another strain-level comparison was not performed. Therefore, clonal identity and the precise route of dissemination could not be established. Third, although both the blood and subdural pus isolates were reported as resistant to colistin, with MICs of ≥16, and *mcr-1* was detected by mNGS in both specimens, the MIC unit, testing platform, and interpretive standard were unavailable, and isolate-level molecular testing was not performed. Therefore, *mcr-1* could not be definitively assigned to either cultured isolate. Fourth, preoperative contrast-enhanced MRI and diffusion-weighted imaging were not obtained because the patient's critical condition made transport unsafe. Finally, although the patient was undergoing further rehabilitation at a rehabilitation hospital with stable vital signs and a GCS score of 15 at the latest follow-up, structured long-term neurological and radiological follow-up data were unavailable, precluding comprehensive assessment of recurrence and long-term functional outcome.

## Conclusion

This case illustrates a high-stakes diagnostic and management pitfall in multidrug-resistant infection. Subdural empyema associated with multidrug-resistant *Escherichia coli* and specimen-level detection of *bla*NDM may closely mimic chronic subdural hematoma on serial non-contrast CT. In the present patient, multidrug-resistant *E. coli* was recovered from both blood and subdural pus, while mNGS detected concordant resistance determinants in both specimens. These findings supported hematogenous dissemination as the most plausible route, although clonal identity was not established. Importantly, apparent clearance of bloodstream infection did not indicate control of the compartmentalized subdural focus. Clinical improvement followed burr-hole drainage combined with antimicrobial therapy, underscoring the decisive role of surgical source control. In patients with bacteremia, persistent fever, neurological deterioration, and an enlarging subdural collection should prompt early reconsideration of subdural empyema despite negative follow-up blood cultures.

## Data Availability

The original contributions presented in the study are included in the article/Supplementary Material, further inquiries can be directed to the corresponding author/s.
