# Supplementary material for: NDM-Producing Escherichia coli subdural empyema mimicking chronic subdural hematoma: a case report
Source: Front Med (Lausanne). 2026 Sep 23;13:1930920. doi: 10.3389/fmed.2026.1930920 (PMC13642217; doi:10.3389/fmed.2026.1930920)
Supplement: Supplementary file 1 [file Table1.docx]

Supplementary Figure S1. Laboratory trends.

*Note:* The upper panel shows C-reactive protein (CRP; solid line with squares) and procalcitonin (PCT; dashed line with triangles). The lower panel shows white blood cell (WBC), neutrophil, lymphocyte, and monocyte counts. Gray dashed vertical lines indicate key clinical events, including admission, positive mNGS results, negative follow-up blood cultures with persistent fever, and burr-hole drainage. The preoperative and postoperative periods are shaded in light orange and light cyan, respectively. Although follow-up blood cultures became negative during days 11–14, fever and elevated inflammatory markers persisted until surgical drainage.
