# Supplementary material for: NDM-Producing Escherichia coli subdural empyema mimicking chronic subdural hematoma: a case report
Source: Front Med (Lausanne). 2026 Sep 23;13:1930920. doi: 10.3389/fmed.2026.1930920 (PMC13642217; doi:10.3389/fmed.2026.1930920)
Supplement: Supplementary file 2 [file Table2.docx]

**Supplementary Material**

**Supplementary Table S1. Microbiological findings and resistance determinants identified by culture and mNGS**

| **Specimen and method** | **Date** | **Organism detected** | **Phenotypic susceptibility** | **Genotypic resistance determinants** |
| --- | --- | --- | --- | --- |
| Blood culture | Mar 4, 2026 | Multidrug-resistant Escherichia coli | Reported as resistant to colistin, with an MIC of ≥16; susceptible to amikacin, minocycline, and tigecycline | Not tested at isolate level |
| Blood mNGS | Reported Mar 6, 2026 | *Escherichia coli* | Not applicable | *bla*NDM (53 reads), *bla*CTX-M (119 reads), and *mcr-1* (137 reads) |
| Subdural pus culture | Mar 22, 2026 | Multidrug-resistant *Escherichia coli*; no yeast-like fungi detected | Reported as resistant to colistin, with an MIC of ≥16; susceptible to amikacin, minocycline, and tigecycline | Not tested at isolate level |
| Subdural pus mNGS | Mar 22, 2026 | *Escherichia coli* | Not applicable | *bla*NDM (1,127 reads), *bla*CTX-M (1,307 reads), and *mcr-1* (2,405 reads) |

*Note:* Phenotypic antimicrobial susceptibility was determined using cultured isolates, whereas resistance genes were detected directly from clinical specimens by mNGS. Because isolate-level molecular testing was not performed, the resistance genes detected by mNGS could not be definitively assigned to the corresponding cultured isolates. For both the blood and subdural pus isolates, the archived laboratory report classified the isolate as resistant to colistin and recorded an MIC of ≥16 but did not provide the MIC unit, testing platform, or interpretive standard. Sequence read counts indicate reads mapped to each resistance gene.

Supplementary Table S2. Clinical course timeline

| **Date** | **Clinical event and findings** | **Treatment or decision** |
| --- | --- | --- |
| Feb 26, 2026 | Admission for fever; chest CT suggested right lower lobe pneumonia; head CT showed a 13-mm left subdural collection | Empirical ceftazidime–avibactam initiated |
| Mar 2, 2026 | Neurological deterioration; ICU transfer with a GCS score of 11; preliminary blood culture showed gram-negative bacilli | Ceftazidime–avibactam changed to meropenem |
| Mar 4, 2026 | Blood culture identified multidrug-resistant *E. coli* | Tigecycline briefly added |
| Mar 5, 2026 | Head CT showed slight enlargement of the subdural collection with decreased density | Continued reassessment; meropenem and tigecycline subsequently discontinued |
| Mar 6, 2026 | Blood mNGS reported *E. coli* and *bla*NDM | Aztreonam–avibactam plus amikacin initiated |
| Mar 13, 2026 | PET-CT did not clarify the nature of the subdural lesion; follow-up blood cultures had become negative, but fever and neurological impairment persisted | Amikacin discontinued and eravacycline initiated |
| Mar 22, 2026 | Head CT showed enlargement of the lesion to 22 mm with cerebral edema; burr-hole exploration revealed yellow purulent material | Evacuation, irrigation, and subdural drainage performed; pus sent for culture and mNGS |
| Mar 23, 2026 | Early postoperative antimicrobial adjustment | Intravenous fosfomycin added |
| Mar 25, 2026 | Postoperative MRI showed residual subdural fluid and meningeal enhancement | Antimicrobial therapy and clinical monitoring continued |
| May 22, 2026 | Completion of prolonged antimicrobial treatment | Aztreonam–avibactam discontinued |
| Latest follow-up | Vital signs were stable, and the GCS score was 15 | Further rehabilitation at a rehabilitation hospital |

*Note:* CT, computed tomography; GCS, Glasgow Coma Scale; ICU, intensive care unit; mNGS, metagenomic next-generation sequencing; MRI, magnetic resonance imaging; PET-CT, positron emission tomography-computed tomography.

Supplementary Table S3. Detailed antimicrobial treatment course

| **Treatment period** | **Antimicrobial regimen** | **Dose, route, and frequency** | **Approximate calendar-day span** | **Rationale for initiation or modification** |
| --- | --- | --- | --- | --- |
| Feb 26–Mar 2, 2026 | Ceftazidime–avibactam | 2.5 g intravenously every 12 h | 5 days | Empirical treatment on admission |
| Mar 2–Mar 5, 2026 | Meropenem | 1 g intravenously every 8 h | 4 days | Empirical escalation after ICU transfer and preliminary detection of gram-negative bacilli. |
| Mar 4–Mar 5, 2026 | Tigecycline | 100 mg intravenous loading dose, followed by 50 mg intravenously every 12 h | 2 days | Added after blood culture identified multidrug-resistant *E. coli*, with a possible gastrointestinal source considered because of previous gastrectomy. |
| Mar 6–May 22, 2026 | Aztreonam–avibactam | 2 g intravenously every 6 h | 78 days | Blood mNGS detection of *E. coli* and *bla*NDM |
| Mar 6–Mar 13, 2026 | Amikacin | 200 mg intravenously every 12 h | 8 days | Reported in vitro susceptibility |
| Mar 13–Mar 23, 2026 | Eravacycline | 50 mg intravenously every 12 h | 11 days | Added as combination therapy because PET-CT showed inflammatory changes around the gastroenteric anastomosis after previous gastrectomy. |
| Mar 23–Mar 27, 2026 | Fosfomycin | 4 g intravenously every 6 h | 5 days | Added after drainage as part of postoperative combination therapy |

*Note:* Calendar-day spans were calculated inclusively from the documented start and stop dates. Postoperative improvement reflected combined surgical source control and antimicrobial therapy rather than fosfomycin alone.
